# Supplementary material for: Do Patients with Complaints Attributed to Chemicals in the Environment Trust in Biomonitoring as a Valid Diagnostic Tool? A Prospective, Observational Study from a German University Outpatient Clinic
Source: Int J Environ Res Public Health. 2025 Jul 18;22(7):1143. doi: 10.3390/ijerph22071143 (PMC12294374; doi:10.3390/ijerph22071143)
Supplement: Supplementary file 1 [file ijerph-22-01143-s001.zip › ijerph-3680046-supplementary.pdf]

Tabelle S1: IPQ-R

IPQ-R	Range	T1 N = 51	T1 N = 33	T1 N = 26 Environmental attribution "stable"	T1 N = 7 Environmental attribution "changed"	T2 N = 26 Environmental attribution "stable"	T2 N = 7 Environmental attribution "changed“	p T1 "stable" vs "changed"	d T1 95-%-CI
Time course	6-30	14.13 (1.64)	13.99 (1.70)	14.20 (1.87)	13.41 (0.72)	14.40 (1.53)	15.36 (1.38)	0.31	-0.462 (-1.304 - 0.38)
Cyclical occurrence	4-20	12.71 (3.15)	13.23 (3.10)	13.5 (3.11)	12.14 (3.02)	12.31 (2.43)	12.29 (2.50)	0.30	-0.44 (-1.281 - 0.402)
Consequences	6-30	16.54 (3.09)	16.60 (3.08)	17.33 (2.90)	14.00 (2.31)	15.97 (2.93)	14.29 (1.80)	0.009	-1.191 (-2.074 - -0.308)
Personal control	6-30	10.99 (3.47)	11.16 (3.26)	11.76 (3.24)	9.00 (2.45)	11.42 (3.70)	10.71 (2.98)	0.046	-0.89 (-1.751 - -0.028)
Treatment control	5-25	12.84 (2.15)	13.22 (1.72)	13.39 (1.82)	12.5 (1.05)	11.67 (2.32)	10.29 (3.20)	0.26	-0.524 (-1.368 - 0.32)
Coherence	5-25	16.62 (3.94)	17.11 (3.63)	17.28 (3.34)	16.5 (4.81)	16.94 (2.86)	16.29 (2.98)	0.62	-0.212 (-1.049 - 0.624)
Emot. Representation	6-30	17.58 (3.34)	18.00 (2.98)	18.31 (2.95)	16.86 (3.02)	17.25 (3.90)	17.14 (2.54)	0.95	-0.489 (-1.332 - 0.354)

Mean values (standard deviations), d = Cohen's d for mean differences between groups of different sizes; CI, confidence interval

Table S2 BSI subscales

	N = 26 Environmental attribution "stable"	N = 7 Environmental attribution "changed"	p "stable" vs "changed"	d 95-%-CI
**Somatisation (7 Items)**	5.96 (4.48)	3.14 (1.07)	0.008	-0.704 (-1.559 - 0.152)
**Compulsiveness (6 Items)**	7.20 (3.43)	3.86 (3.44)	0.03	-0.976 (-1.848 - -0.105)
**Insecurity in social contact (4 Items)**	2.28 (1.77)	1.43 (0.98)	0.11	-0.515 (-1.362 – 0.333)
**Depression (6 Items)**	4.60 (2.60)	2.43 (3.10)	0.07	-0.804 (-1.665 – 0.057)
**Anxiety (6 Items)**	4.44 (2.81)	4.86 (2.85)	0.73	0.145 (-0.693 – 0.984)
**Aggressiveness/hostility (5 Items)**	4.20 (2.53)	2.57 (1.51)	0.13	-0.673 (-1.528 – 0.181)
**Phobic fear (5 Items)**	3.24 (2.71)	2.00 (1.41)	0.26	-0.495 (-1.342 – 0.352)
**Paranoid thinking (5 Items)**	3.48 (1.78)	2.85 (1.86)	0.43	-0.345 (-1.188 – 0.497)
**Psychoticism (5 Items)**	3.72 (2.37)	2.00 (2.45)	0.10	-0.721 (-1.577 – 0.136)

Mean values ± standard deviations
